# Generative AI–Assisted Simulation Training Is Associated with Higher Post-Intervention Diagnostic Communication Scores Across Type 2 Diabetes, Obesity, and Breast Cancer Scenarios

**DOI:** 10.3390/healthcare14131883

**Published:** 2026-06-28

**Authors:** Bruno Manuel García-García, Bguelly Jean N’guessan-Sánchez, María Fernanda Romero-Guevara, Jazel Jarquín-Ramírez, Nallely Guadalupe Aguilar-Marchand, María Guadalupe Gutiérrez-López, César Javier Sánchez-Ramón, Ari Evelyn Castañeda-Ramírez, Angel Corchado-Vargas, Pável Eber Bautista Portilla, Ángel Elizalde-Méndez, Isis Villafuerte-Tunaal, Adolfo René Méndez-Cruz, Brenda Ofelia Jay-Jímenez, Héctor Iván Saldívar-Cerón

**Affiliations:** 1 Carrera de Médico Cirujano, Facultad de Estudios Superiores-Iztacala, Universidad Nacional Autónoma de México, Tlalnepantla 54090, Mexico; bruno.garcia12@comunidad.unam.mx (B.M.G.-G.); 317588576@iztacala.unam.mx (B.J.N.-S.); fernanda19.romero.g@gmail.com (M.F.R.-G.); jazel.jarquin@iztacala.unam.mx (J.J.-R.); nallely.marchand@iztacala.unam.mx (N.G.A.-M.); dra.gutierrez@iztacala.unam.mx (M.G.G.-L.); bvbcesarfcb@gmail.com (C.J.S.-R.); aricasra206@gmail.com (A.E.C.-R.); armendez@unam.mx (A.R.M.-C.); 2 Unidad de Remisión de Diabetes Mellitus (URDM), Facultad de Estudios Superiores-Iztacala, Universidad Nacional Autónoma de México, Tlalnepantla 54090, Mexico; 3 Centro Internacional de Simulación y Entrenamiento en Soporte Vital Iztacala (CISESVI), Facultad de Estudios Superiores-Iztacala, Universidad Nacional Autónoma de México, Tlalnepantla 54090, Mexico; 4 Carrera de Psicología, Facultad de Estudios Superiores-Iztacala, Universidad Nacional Autónoma de México, Tlalnepantla 54090, Mexico; angel.corchado@iztacala.unam.mx; 5 Departamento de Farmacia, Facultad de Quimica, Universidad Nacional Autónoma de México, Mexico City 04510, Mexico; pavel.ebp06@gmail.com; 6 Servicio de Ginecología Oncológica, Instituto Nacional de Perinatología, Mexico City 11000, Mexico; gineco.oncologo.angelelizalde@gmail.com; 7 Facultad de Medicina, Universidad Autónoma de Durango, Mazatlan 82123, Mexico; isisvillafuertet@gmail.com

**Keywords:** medical education, undergraduate medical education, diagnostic communication, generative artificial intelligence, large language models, simulation training, standardized patients, virtual patients, type 2 diabetes mellitus, obesity, breast cancer

## Abstract

**Background**: Diagnostic communication influences patient understanding, adherence, and shared decision-making in high-burden cardiometabolic disease and high-stakes oncologic care. However, scalable training models that allow standardized, repeatable practice and competency benchmarking remain limited. This study examined whether undergraduate medical students demonstrated higher diagnostic communication scores after completing a structured generative artificial intelligence (AI)-assisted simulation program across three clinically distinct diagnostic disclosure scenarios. **Methods**: We conducted a prospective, single-arm, pre–post educational study in undergraduate medical students completing AI-assisted diagnostic communication training across T2DM, obesity, and breast cancer scenarios. Students underwent baseline in-person assessments with standardized human simulated patients, completed 10 asynchronous AI-assisted encounters per scenario using standardized scenario-specific prompts and automated feedback, and then completed post-intervention in-person assessments. Scenario order was randomized. Performance was scored live by two physician raters using an adapted 24-item, eight-domain rubric. Cross-scenario analyses included three-scenario completers (*n* = 56; scenario-specific paired samples up to *n* = 77). Without a control group, analyses were interpreted as within-student pre–post associations rather than causal effects. **Results**: Students demonstrated higher post-test total rubric scores across all scenarios. Mean (SD) within-student changes were +24.26 (25.05) for T2DM, +26.17 (20.67) for obesity, and +36.31 (17.70) for breast cancer. Positive pre–post changes were observed across communication domains, with variation by clinical context. Exploratory analyses suggested limited cross-scenario gain-score associations and heterogeneous response patterns. **Conclusions**: Generative AI-assisted simulation was associated with higher post-intervention diagnostic communication scores across three diagnostic disclosure scenarios. The single-arm design precludes causal attribution and does not exclude testing effects, rubric familiarization, maturation, or concurrent clinical learning. Controlled studies are needed to determine its comparative educational value.

## 1. Introduction

Diagnostic communication is a core clinical competency in medical practice. The manner in which clinicians disclose a diagnosis shapes patient understanding, emotional processing, trust, adherence, and engagement with subsequent care. In chronic cardiometabolic diseases such as type 2 diabetes mellitus (T2DM) and obesity, these processes influence whether patients adopt and sustain the therapeutic behaviors required for long-term disease control. In oncology, where diagnostic disclosure often carries greater emotional weight, communication quality also affects distress, prognostic understanding, and shared decision-making [1,2,3,4].

These considerations make diagnostic communication highly relevant to both educational quality and patient safety. Communication failures have been repeatedly identified as important contributors to adverse events, handoff problems, and malpractice claims, supporting the need for structured training rather than reliance on informal experiential learning alone [5,6]. As competency-based medical education increasingly emphasizes observable and benchmarkable performance, diagnostic communication requires training models that are standardized, reproducible, and scalable.

However, scalable training in diagnostic disclosure remains difficult to implement. Standardized patients and other face-to-face simulation modalities offer substantial educational value, but they are resource-intensive and difficult to repeat at high frequency across large student cohorts. Virtual patient strategies have therefore gained attention as a means of expanding deliberate practice while preserving standardization and assessment opportunities [7]. Generative artificial intelligence (AI) may complement these approaches by enabling dynamic, open-ended dialogue and individualized formative feedback, although its added educational value over established simulation modalities requires empirical evaluation.

Recent advances in large language models (LLMs) have further increased the feasibility of interactive virtual patients capable of generating dynamic, context-sensitive dialogue. Recent systematic reviews document the rapid expansion of ChatGPT (GPT-5) and related LLM applications in healthcare education and medicine, with recurring interest in tutoring, simulation, feedback, and communication training [8,9,10]. At the same time, these reviews also highlight important limitations, including hallucinations, inconsistency, bias, and unresolved governance concerns [8,9,10]. Accordingly, the educational value of AI-assisted simulation should be established through rigorous outcome-based evaluation rather than inferred from novelty alone.

A key educational question is whether post-training communication score changes are consistent across clinically distinct diagnostic disclosure contexts. Many studies evaluate a single scenario and may implicitly treat performance in one case type as a proxy for broader communicative competence. Yet diagnostic disclosure is not a uniform task. Communicating T2DM, counseling a patient with obesity, and disclosing a breast cancer diagnosis differ in explanatory demands, emotional intensity, stigma burden, uncertainty, perceived threat, and the balance required between biomedical information delivery and empathic support [11,12]. These differences provide a rationale for evaluating multiple scenarios rather than assuming that performance changes in one diagnostic context necessarily generalize to another.

A second challenge is the marked heterogeneity of learner response. Mean pre–post score changes may obscure important variability in how students perform across domains and scenarios, which is directly relevant to competency-based assessment, targeted remediation, and curricular design. In our initial DIALOGUE study, a single-group evaluation suggested that generative AI-assisted simulation was associated with higher post-training diagnostic communication performance in a T2DM disclosure scenario [13]. A subsequent randomized controlled trial comparing conventional diagnostic communication training with conventional training supplemented by AI-supported adaptive simulation also supported the feasibility of this approach, although baseline-adjusted group-by-time interactions did not reach conventional thresholds for statistical significance and the added mean benefit beyond conventional training remained uncertain [14]. Together, these findings support further evaluation of AI-assisted simulation while underscoring that its educational contribution should be interpreted cautiously and examined across different clinical communication contexts rather than inferred from a single diagnostic scenario.

The present study, therefore, extended this line of work from a single T2DM disclosure scenario to three clinically distinct diagnostic contexts: T2DM, obesity, and breast cancer. We aimed to examine whether completion of a structured generative AI-assisted simulation curriculum was associated with higher post-intervention diagnostic communication scores across scenarios, whether the magnitude of pre–post score change differed by clinical context, and whether exploratory gain-score analyses suggested cross-scenario associations or inter-individual heterogeneity in response patterns.

## 2. Materials and Methods

### 2.1. Study Design, Educational Context, and Ethical Approval

This was a prospective, single-arm, pre–post educational intervention study designed to evaluate the association between generative artificial intelligence (AI)-assisted simulation training and diagnostic communication performance across three clinically distinct diagnostic disclosure scenarios: type 2 diabetes mellitus (T2DM), obesity, and breast cancer. Because all participants received the intervention and no no-intervention, wait-list, active comparator, or non-AI simulation control group was included, the study was not designed to establish causal effectiveness. Observed pre–post changes were therefore interpreted as within-student associations after curriculum completion rather than as causal effects of the AI-assisted intervention. Potential alternative explanations included repeated exposure to the assessment format, maturation, concurrent clinical learning, rubric familiarization, testing effects, and regression to the mean. The study was conducted between September and October 2025 at the Facultad de Estudios Superiores Iztacala (FES Iztacala), Universidad Nacional Autónoma de México (UNAM), Mexico. The study protocol was finalized before participant enrollment, and no changes to the intervention sequence, assessment framework, or analytic strategy were introduced after study initiation.

Because the study comprised three scenario-based evaluations corresponding to distinct clinical communication contexts, separate ethics approvals and informed consent procedures were obtained before implementation. The study was approved by the Institutional Ethics Committee of FES Iztacala, UNAM, under protocol codes CE/FESI/052025/1922 (May 2025), CE/FESI/082025/1988 (August 2025), and CE/FESI/092025/2006 (September 2025). Written informed consent was obtained from all participants before enrollment and before participation in each of the three scenario-based evaluations.

The study was embedded within an educational activity of a comprehensive clinical course conducted during students’ clinical rotations. However, participation in the research component was voluntary. Students were explicitly informed that participation, non-participation, or withdrawal would have no effect on their academic standing, course performance, or relationship with faculty. No financial incentives were provided. The study was conducted in accordance with the Declaration of Helsinki.

### 2.2. Participants and Recruitment

Participants were undergraduate medical students enrolled in the clinical-cycle curriculum at FES Iztacala, UNAM. Students in the clinical cycles were invited to participate during a course-related educational activity conducted within their clinical rotations. Participants were recruited through voluntary convenience sampling from the eligible clinical-cycle student population.

The number of eligible students invited, enrolled students, scenario-specific completers, and three-scenario completers is reported in the participant flow diagram. Because recruitment was voluntary and convenience-based, selection bias was considered possible, particularly in relation to motivation, digital self-efficacy, and prior LLM use.

Eligibility criteria were active enrollment in the clinical-cycle curriculum and availability to complete both the in-person pre-test and post-test assessments. Exclusion criteria were failure to complete baseline assessment procedures or failure to provide written informed consent. All assessments were conducted individually under standardized on-campus conditions. When available, baseline characteristics of three-scenario completers and non-completers were compared descriptively to assess potential attrition-related bias.

### 2.3. Pre-Test Assessment

Baseline diagnostic communication performance was evaluated through an in-person pre-test conducted on campus under standardized conditions. Each assessment consisted of a live diagnostic disclosure encounter in one of three predefined clinical scenarios: T2DM, obesity, or breast cancer.

Across scenarios, students were required to perform the core components of a diagnostic communication encounter, including establishing rapport and agenda, eliciting the patient’s perspective, delivering diagnostic information clearly, responding empathically to emotional reactions, and closing the encounter with an appropriate management plan and follow-up orientation. Pre-test encounters were conducted with previously trained standardized human simulated patients. Simulated patients underwent prior preparation to ensure consistent portrayal of the clinical scenario, communicative demands, and expected emotional responses. All encounters were scored live. Because encounters were scored live rather than from anonymized video recordings, complete blinding to the assessment phase could not be guaranteed.

### 2.4. AI-Assisted Simulation Training

After completion of the pre-test, all enrolled participants completed an asynchronous simulation-based training program focused on diagnostic communication. The intervention was delivered using GPT-5 through the ChatGPT interface (OpenAI, San Francisco, CA, USA). GPT-5 had been officially released by OpenAI on 7 August 2025, before the September–October 2025 study implementation period. No custom fine-tuning was performed. The model was configured through standardized scenario-specific prompt templates developed a priori by the research team. Because the intervention was implemented through the ChatGPT interface rather than a fully controlled API environment, model-level parameters such as temperature, top-p, sampling seed, and backend routing were not controlled by the investigators; this limitation is acknowledged in relation to reproducibility.

Three scenario-specific standardized prompt templates were developed a priori by the research team, one for each clinical context: T2DM, obesity, and breast cancer. These prompts were designed to preserve scenario fidelity, standardize the communicative task, and maintain comparable virtual patient behavior across participants within each scenario. Each prompt instructed the model to act as a standardized virtual patient, maintain the assigned clinical and emotional profile, avoid providing expert-level medical guidance to the learner during the encounter, and generate structured formative feedback only after completion of the simulated diagnostic disclosure. Feedback was organized around the adapted Kalamazoo domains and included strengths, missed opportunities, and suggestions for subsequent practice.

Each participant completed 10 AI-assisted training encounters per scenario, for a total of 30 simulation sessions across the intervention. During each session, participants were instructed to communicate the diagnosis in a realistic and professionally appropriate manner, address patient concerns and emotional cues, and iteratively incorporate the feedback generated by the model into subsequent encounters. Formative feedback was generated automatically by ChatGPT after each simulated interaction. Completion of the 30 encounters was verified using exported chat records. Students were allowed to repeat sessions for additional practice, but only the required 10 encounters per scenario were counted for completion. Students were instructed not to use external resources during asynchronous practice; this was considered when interpreting the intervention’s ecological validity.

To address privacy and data protection, students were instructed not to enter personal health information, identifiable patient data, or confidential third-party information into the platform. All simulated cases were fictional and contained no real patient identifiers. Participants were informed that AI outputs could be incomplete, inconsistent, or inaccurate and that model-generated feedback was formative rather than authoritative clinical guidance. Prompt templates and representative outputs were reviewed by faculty before implementation to assess scenario fidelity, clinical appropriateness, empathic tone, and potential hallucination or bias. Individual student-level AI outputs were not exhaustively audited in real time, which was recorded as a limitation.

To reduce potential sequence effects and rubric familiarization bias, the order of scenario exposure was randomized across participants using a computer-generated simple random sequence. Thus, participants initiated training with different scenarios and subsequently completed the remaining ones. Although the intervention was asynchronous, all participants completed the same three-scenario training structure during the study period. Prompt templates, simulation materials, scoring materials, and training resources are publicly available in the Open Science Framework repository listed in the Data Availability Statement.

### 2.5. Post-Test Assessment

Following completion of the AI-assisted training phase, participants underwent an in-person post-test conducted on campus under standardized conditions. As in the baseline phase, each post-test consisted of a live diagnostic disclosure encounter in one of the three predefined clinical scenarios: T2DM, obesity, or breast cancer.

The communicative tasks required during post-test encounters were equivalent to those assessed at baseline, including rapport building, elicitation of the patient’s perspective, clear explanation of the diagnosis, empathic response to emotional reactions, and closure with an appropriate plan and follow-up orientation. Post-test encounters were conducted with previously trained standardized human simulated patients using preparation procedures analogous to those applied during the pre-test phase. All post-test encounters were scored live. Standardized patients were trained to portray the same clinical and emotional profile across participants within each scenario. Standardized patients were blinded to assessment timing and were not involved in scoring.

### 2.6. Outcome Measures and Scoring Instrument

The primary outcome was within-student change in diagnostic communication performance from pre-test to post-test, defined as Δ = post-test − pre-test in the total rubric score for each scenario. Secondary outcomes included pre–post changes in each of the eight communication domains, exploratory associations between scenario-specific gain scores, and inter-individual heterogeneity in learning trajectories assessed through domain-level change profiles and exploratory clustering procedures.

Performance was assessed using an adapted version of the Kalamazoo Essential Elements Communication Checklist, originally described as a seven-domain framework for communication assessment in medical encounters [15]. In the present study, the rubric was adapted to the context of diagnostic disclosure and operationalized as 24 items distributed across eight domains, with three items per domain. The first seven domains were based on the original Kalamazoo framework, whereas an eighth domain—Scenario-Specific Communication—was developed by the research team to capture communicative competencies specific to the diagnostic demands of each clinical scenario. Each item was rated on a 5-point Likert scale. Domain scores were calculated as the sum of the three constituent items (range, 3–15), and the total score was calculated as the sum of all 24 items (range, 24–120), with higher scores indicating better communication performance. The eight domains were: (1) Build a Relationship, (2) Opening the Discussion, (3) Gather Information, (4) Understand the Patient’s Perspective, (5) Share Information, (6) Reach Agreement, (7) Provide Closure, and (8) Scenario-Specific Communication. The full adapted 24-item rubric, item-level scoring anchors, scenario-specific communication items, and scoring materials are publicly available in the OSF repository listed in the Data Availability Statement. The adaptation process included expert review by faculty with experience in medical education, simulation, endocrinology/metabolic disease, oncology/gynecology, and psychology. The adapted instrument was reviewed for content relevance, clarity, scenario fit, and rater usability before implementation.

### 2.7. Blinding, Rater Calibration, and Scoring Procedures

All live encounters were independently evaluated by two physician raters with prior teaching experience in medical education. Raters did not participate in the delivery of the intervention and were kept unaware of participants’ exposure to the AI-assisted training program. Specifically, they were not informed that students had completed a generative AI-supported simulation curriculum before the post-test assessments. However, because encounters were scored live and not from anonymized recordings presented in random order, complete blinding to the pre-test versus post-test phase could not be guaranteed. This was considered a potential source of expectation bias.

The same rubric, scoring anchors, and calibration procedures were used across scenarios and time points. The same physician raters did not score both pre-test and post-test encounters. Raters could not identify participants by name or previous encounter. Scenario order assignment was not concealed from raters. Before study initiation, evaluators underwent rubric training and calibration to standardize scoring criteria across scenarios and time points. Inter-rater reliability was assessed using weighted Cohen’s kappa at the domain level. Inter-rater agreement was >0.80, supporting acceptable scoring consistency for rubric-based live assessment. Disagreements between raters were resolved by third-rater review, and final analytic scores were computed using adjudicated scores. For continuous domain and total scores, inter-rater reliability was additionally evaluated using intraclass correlation coefficients (ICCs) with 95% confidence intervals.

### 2.8. Statistical Analysis

Analyses were performed at two complementary levels. First, scenario-level analyses included all participants with complete paired pre-test and post-test data for the corresponding scenario. Second, cross-scenario analyses were restricted to the complete-case subgroup of students with paired pre-test and post-test data across all three scenarios.

Continuous variables are presented as mean ± standard deviation (SD) or median and interquartile range (IQR), as appropriate. Categorical variables are presented as frequencies and percentages. For each scenario, pre-test and post-test total scores and domain scores were compared using paired *t*-tests. Within-student change was summarized as Δ = post-test − pre-test. Paired effect sizes were estimated using Cohen’s dz, calculated as the mean of the paired differences divided by the standard deviation of those paired differences. Ninety-five percent confidence intervals (95% CIs) were reported where applicable. To control the family-wise error rate in domain-level analyses, *p*-values were adjusted using the Holm procedure.

As a sensitivity analysis, a linear mixed-effects model was fitted to evaluate total rubric scores across time and scenario while accounting for repeated observations within students. The model included fixed effects for time, scenario, and the time-by-scenario interaction, with a random intercept for student. The time-by-scenario interaction was used to examine whether the magnitude of pre–post score change differed across T2DM, obesity, and breast cancer scenarios. Estimated marginal means and pairwise contrasts of scenario-specific pre–post changes were reported with multiplicity adjustment. If model assumptions were not met, results were interpreted cautiously and compared with the paired *t*-test findings.

For the analysis of average within-student gains across the subgroup of participants who completed all three scenarios, one-sample *t*-tests were used to test whether mean Δ differed from 0 for each domain and for the total score, with Holm correction applied across the nine outcomes. To explore baseline–gain relationships, associations between baseline pre-test scores and Δ values were examined using regression-based visualization. Because gain scores are sensitive to baseline scores, regression to the mean, ceiling effects, and measurement error, these analyses were interpreted cautiously. Where feasible, residualized change scores from baseline-adjusted models were also examined as an exploratory sensitivity analysis.

Because gain-score distributions were not assumed to be strictly Gaussian and cross-scenario associations were treated as exploratory rank-based constructs, associations between scenario-specific Δ values were evaluated using Spearman correlation coefficients. Multiple cross-scenario comparisons were interpreted cautiously because of the small number of pairwise tests and the exploratory nature of the analysis.

To characterize heterogeneity in learning trajectories, exploratory clustering analyses were performed on domain-level and scenario-level change profiles. For heatmap visualization, domain-level Δ scores were standardized within each scenario using z-scores to emphasize relative change patterns across domains. Students were grouped using k-means clustering with k = 3, selected as a pragmatic and interpretable solution for identifying exploratory response patterns across scenarios. Because clusters were derived from the same gain variables used to describe them, between-cluster differences were presented descriptively and were not interpreted as independent inferential evidence. The k = 3 solution was selected a priori as a pragmatic descriptive grouping to aid visualization and was not intended as a statistically optimized or validated clustering solution.

Baseline characteristics of three-scenario completers and non-completers were compared descriptively using *t*-tests, Mann–Whitney U tests, chi-square tests, or Fisher’s exact tests as appropriate. These analyses were exploratory and intended to assess potential attrition bias rather than to support causal inference. All tests were two-sided, and statistical significance was defined as *p* < 0.05. All analyses were performed in R using RStudio (version 2025.05.1+513).

## 3. Results

### 3.1. Participant Flow and Baseline Characteristics

**Figure 1:**
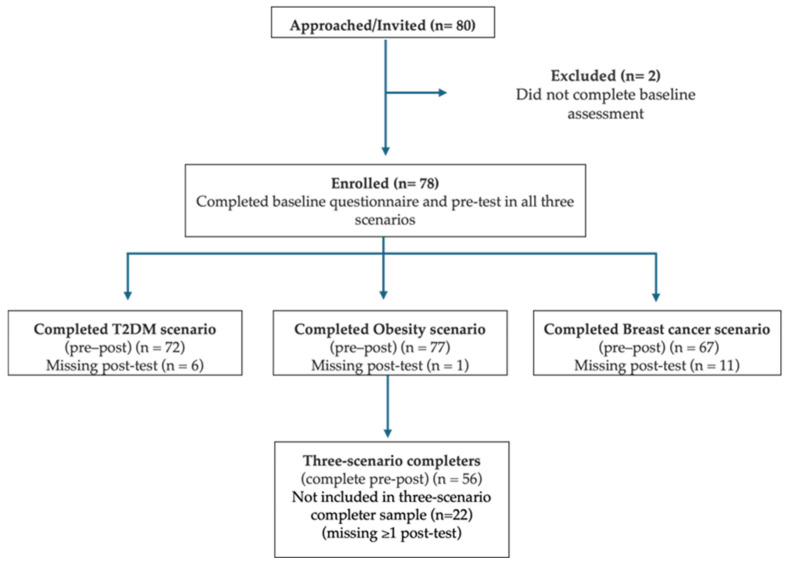
Participant flow diagram and analytic samples for three diagnostic communication scenarios.

A total of 80 students were invited to participate, of whom 78 completed enrollment and baseline pre-test assessments. Scenario-specific post-test completion yielded analytic samples with complete paired rubric scores of 72 students for T2DM, 77 for obesity, and 67 for breast cancer. Cross-scenario analyses were restricted to the 56 students who completed both pre-test and post-test assessments across all three scenarios, allowing within-student comparisons across clinical contexts (Figure 1).

Baseline characteristics of the three-scenario completer subgroup are summarized in Table 1. Participants had a mean age of 21.9 ± 1.5 years, 71.4% were women, and all were enrolled in Cycle III of the clinical curriculum. No participant reported prior formal coursework in diagnostic communication. Prior unsupervised real-patient care and prior communication of weight-, obesity-, or metabolic-risk results were each reported by 69.6% of students. Prior use of ChatGPT or other large language models was common, although the frequency of use varied. Motivation to participate, digital self-efficacy, communication-related self-confidence, and empathy scores are presented descriptively in Table 1 to characterize the analytic sample. Baseline characteristics of three-scenario completers and non-completers were compared descriptively to assess potential attrition-related bias. No major baseline imbalances were observed in the variables available for comparison; however, because 22 enrolled students did not complete all three post-test assessments, attrition-related selection bias cannot be excluded.

### 3.2. Pre–Post Changes in Overall Diagnostic Communication Performance

**Figure 2:**
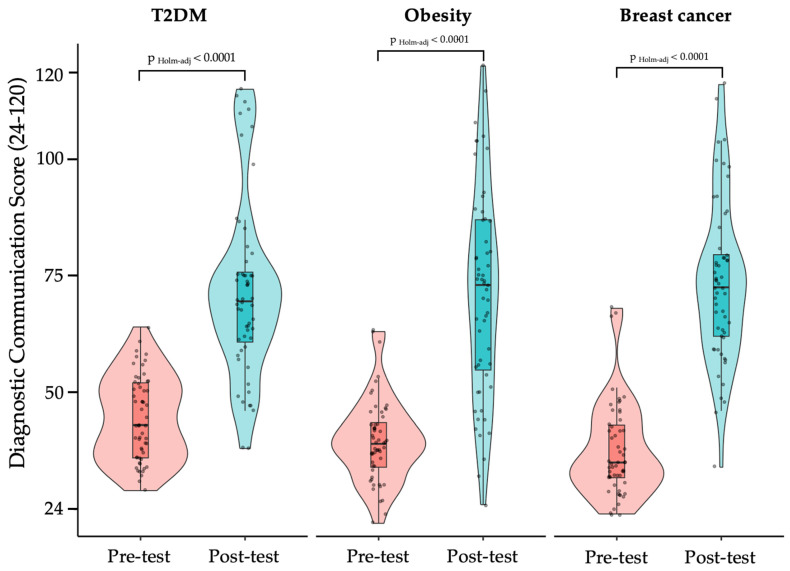
Pre-test and post-test total diagnostic communication scores across three clinical scenarios (T2DM, obesity, and breast cancer) among students completing all scenarios (*n* = 56). Pre-test and post-test distributions of total diagnostic communication scores (range 24–120) across three clinical scenarios (T2DM, obesity, and breast cancer) in medical students who completed all three scenarios (*n* = 56). Each dot represents an individual student; the same students contribute to both pre- and post-test observations within each scenario. The colored violin areas represent the distribution density of scores within each group, while the em-bedded boxplots summarize the median and interquartile range (IQR). Statistical comparisons were performed within each scenario using paired *t*-tests, with Holm adjustment applied across the three scenario-level comparisons. For all scenarios, post-test scores were significantly higher than pre-test scores (Holm-adjusted *p* < 0.0001). These comparisons describe within-student pre–post score differences and do not establish causal effects.

Students demonstrated higher post-test total rubric scores across all three clinical scenarios. As a sensitivity analysis, a linear mixed-effects model was fitted using total rubric score as the dependent variable, with fixed effects for time, scenario, and the time-by-scenario interaction, and a random intercept for student. The model showed a positive time effect across scenarios, indicating higher post-test scores overall. The time-by-scenario interaction was statistically significant, supporting scenario-dependent differences in the magnitude of pre–post score change. Estimated marginal contrasts showed that the breast cancer scenario had a larger pre–post score change than both T2DM and obesity. The estimated difference in pre–post change was +12.05 points for breast cancer versus T2DM and +10.14 points for breast cancer versus obesity. In contrast, the difference between obesity and T2DM was small (+1.91 points) and did not indicate a meaningful scenario-level difference. These model-based findings were consistent with the paired analyses and support interpreting breast cancer as the scenario with the largest descriptive and model-supported pre–post score change, while maintaining the non-causal interpretation required by the single-arm design. As shown in Figure 2, total rubric score distributions shifted upward from pre-test to post-test in T2DM, obesity, and breast cancer, with the largest descriptive absolute change observed in the breast cancer scenario. Paired comparisons showed statistically significant pre–post score differences in each scenario after Holm adjustment for the three scenario-level tests. Mean (SD) within-student changes in total score were +24.26 (25.05) for T2DM, +26.17 (20.67) for obesity, and +36.31 (17.70) for breast cancer. These findings indicate large within-student pre–post score increases across cardiometabolic and oncologic disclosure contexts; however, because of the single-arm design, they should not be interpreted as causal effects of the AI-assisted intervention.

### 3.3. Domain-Level Pre–Post Communication Performance

Pre-test and post-test domain scores across the three scenarios are presented in Table 2. Post-test domain scores were higher than pre-test scores across all eight communication domains within each clinical context. Although the direction of pre–post change was consistently positive, the magnitude of score change varied across both domains and scenarios. In descriptive terms, larger score changes were concentrated in domains related to encounter structuring and information exchange, whereas changes in more complex patient-centered processes were generally smaller and more variable. This pattern was especially evident in the breast cancer scenario, in which post-test values exceeded pre-test values across all domains, with particularly marked changes in Build a Relationship, Opening the Discussion, Gather Information, Share Information, and Provide Closure. Similar, although less pronounced, patterns were observed in T2DM and obesity.

### 3.4. Within-Student Domain-Level Gains

Within-student change estimates for each communication domain are summarized in Table 3. Across scenarios, score changes were consistently positive, although their magnitude varied by both domain and clinical context.

In the T2DM scenario, the largest mean positive score changes were observed in Opening the Discussion (Δ = 4.17 ± 3.33), Provide Closure (Δ = 3.43 ± 3.99), and Share Information (Δ = 3.39 ± 4.09), whereas smaller changes were observed in Understand the Patient’s Perspective (Δ = 2.17 ± 4.27), Build a Relationship (Δ = 2.60 ± 3.77), and Reach Agreement (Δ = 2.61 ± 3.72). In the obesity scenario, the largest mean positive score changes were observed in Share Information (Δ = 3.75 ± 3.43), Scenario-Specific Communication (Δ = 3.52 ± 3.51), and Build a Relationship (Δ = 3.38 ± 3.00), whereas comparatively smaller changes were observed in Understand the Patient’s Perspective (Δ = 2.77 ± 3.12) and Reach Agreement (Δ = 3.09 ± 3.35). In the breast cancer scenario, positive score changes were generally larger across domains, particularly for Gather Information (Δ = 6.37 ± 2.76), Build a Relationship (Δ = 4.97 ± 2.98), Provide Closure (Δ = 4.94 ± 2.83), Share Information (Δ = 4.85 ± 3.12), and Opening the Discussion (Δ = 4.61 ± 2.92).

Among students who completed all three scenarios, average within-student score changes across scenarios are summarized in Table 4. All eight domains showed statistically significant positive pre–post score changes after Holm correction. The largest average changes were observed in Gather Information (Δ = 4.15 ± 1.75, 95% CI 3.69–4.62, dz = 2.38), Opening the Discussion (Δ = 4.05 ± 1.77, 95% CI 3.58–4.53, dz = 2.29), and Share Information (Δ = 4.03 ± 1.97, 95% CI 3.50–4.56, dz = 2.04). The smallest positive pre–post score change was observed in Understand the Patient’s Perspective (Δ = 2.26 ± 2.00, 95% CI 1.73–2.80, dz = 1.13). Scenario-Specific Communication also showed a statistically significant positive pre–post score change (Δ = 3.58 ± 2.12, 95% CI 3.02–4.15, dz = 1.69). At the total-score level, the mean overall change averaged across scenarios was +28.68 ± 11.66 (95% CI 25.56–31.81; dz = 2.46; Holm-adjusted *p* < 0.0001).

**Figure 3:**
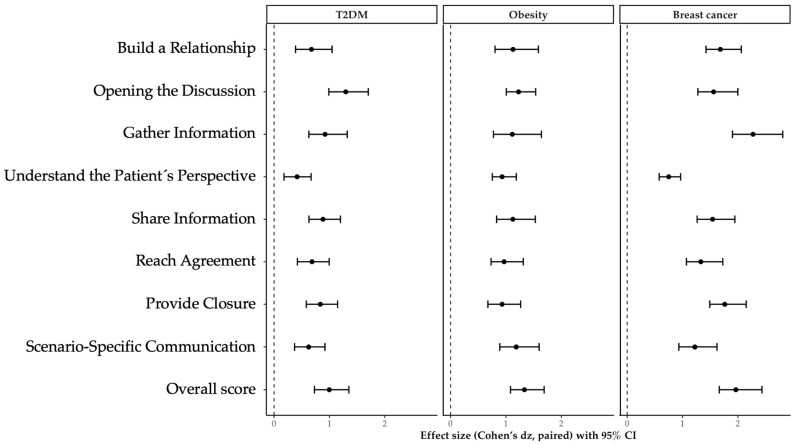
Forest plot of within-student effect sizes (Cohen’s dz) across diagnostic communication domains and overall performance, stratified by clinical scenario (T2DM, obesity, and breast cancer). Effect sizes quantify the magnitude of within-student pre–post score change for each domain. Points indicate the paired effect size and horizontal lines the corresponding 95% confidence intervals; the vertical dashed line marks no change (dz = 0). Across scenarios, effect sizes were consistently positive, with the largest values clustering in the breast cancer scenario, particularly for information gathering and the overall score, whereas the smallest effects were observed in “Understand the Patient’s Perspective”. Analyses were restricted to students with complete paired data across all three scenarios (*n* = 56).

These domain-level patterns were mirrored in the standardized effect-size profiles shown in Figure 3. Across all three scenarios, effect sizes were consistently positive, with the largest values generally observed in the breast cancer scenario, particularly for Gather Information and overall performance. By contrast, smaller—although still positive—effects were observed in domains related to understanding the patient’s perspective. Overall, these findings indicate broad positive pre–post score changes across communication domains, with larger changes concentrated in information exchange and encounter organization rather than in the more complex domain of understanding the patient’s perspective.

### 3.5. Baseline Performance and Gain-Score Relationships

**Figure 4:**
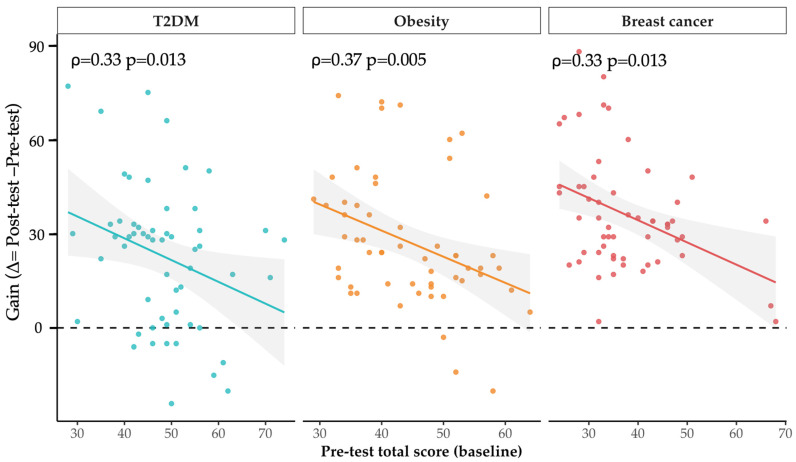
Baseline performance vs. pre–post score change across three diagnostic communication scenarios (*n* = 56). Association between baseline performance and pre–post score change in diagnostic communication across three clinical scenarios (T2DM, obesity, and breast cancer) among students who completed all scenarios (*n* = 56). Each point represents one student. The x-axis shows the pre-test total rubric score, and the y-axis shows the gain score (Δ = post-test − pre-test). Different colors represent the three clinical scenarios: T2DM, obesity, and breast cancer. Solid colored lines depict the fitted linear trend within each scenario, and the grey shaded areas represent the corresponding 95% confidence bands. The dashed horizontal line at Δ = 0 indicates no change. The negative slope in each panel illustrates a consistent pattern whereby students with higher baseline scores tended to show smaller absolute pre–post score changes, whereas students with lower baseline scores tended to show larger absolute pre–post score changes. These associations are descriptive and should be interpreted cautiously because gain scores are sensitive to baseline performance, regression to the mean, ceiling effects, and measurement error.

Baseline performance was inversely associated with the magnitude of pre–post score change across all three scenarios. As shown in Figure 4, students with lower pre-test scores tended to exhibit larger absolute score changes, whereas those with higher baseline scores showed more attenuated changes. This pattern was observed consistently in T2DM, obesity, and breast cancer. Because baseline–gain analyses are inherently sensitive to mathematical coupling, regression to the mean, ceiling effects, and measurement error, these associations should be interpreted cautiously. The visual pattern suggests non-uniform pre–post score change across the baseline performance spectrum, but it should not be interpreted as evidence that the intervention had a larger causal effect among lower baseline performers. Given the exploratory nature of the study and the limited complete-case sample size for three-scenario analyses, additional multivariable predictor modeling was not performed. Baseline characteristics potentially related to learning response, including prior ChatGPT/LLM use, digital self-efficacy, empathy, communication-related self-confidence, GPA, prior real-patient care, and prior diagnostic communication experience, are reported descriptively in Table 1 to characterize the analytic sample rather than to support inferential prediction of score change.

### 3.6. Exploratory Cross-Scenario Associations of Gain Scores

**Figure 5:**
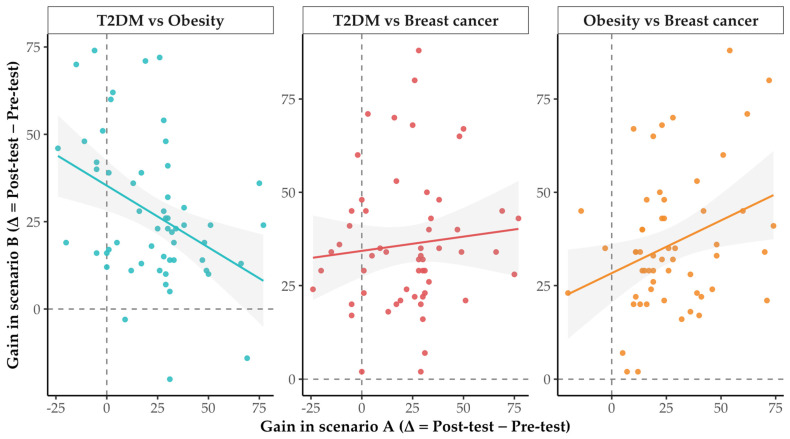
Exploratory cross-scenario associations of gain scores across three diagnostic communication scenarios. Scatterplots relate each student’s gain score (Δ = post-test − pre-test total score) between pairs of scenarios: T2DM vs. obesity, T2DM vs. breast cancer, and obesity vs. breast cancer (*n* = 56 students completing all three scenarios). Each point represents one student. Different colors distinguish the pairwise scenario comparisons. Solid lines show the fitted linear trend for each comparison, and the grey shaded areas represent the corresponding 95% confidence bands. Dashed reference lines at Δ = 0 indicate no score change; points in the upper-right quadrant reflect positive pre–post score changes in both scenarios, whereas points in opposite quadrants reflect scenario-specific score changes. Spearman’s ρ and *p*-values summarize rank-based associations of gain scores between scenarios. These analyses were exploratory and should not be interpreted as direct evidence of transfer or lack of transfer across diagnostic contexts.

Exploratory cross-scenario gain-score associations were limited and context-dependent. As shown in Figure 5, gain scores were inversely correlated between T2DM and obesity (Spearman’s ρ = −0.38, *p* = 0.004), were not significantly associated between T2DM and breast cancer (ρ = 0.08, *p* = 0.55), and were weakly positively correlated between obesity and breast cancer (ρ = 0.27, *p* = 0.047). These exploratory correlations should not be interpreted as direct evidence of transfer or lack of transfer across diagnostic contexts. Gain-score correlations are sensitive to baseline performance, regression to the mean, ceiling effects, and measurement error. Taken together, the observed pattern suggests that pre–post score changes in one scenario did not consistently correspond to pre–post score changes in another scenario, supporting the rationale for multi-scenario assessment.

### 3.7. Exploratory Heterogeneity of Pre–Post Change Patterns

**Figure 6:**
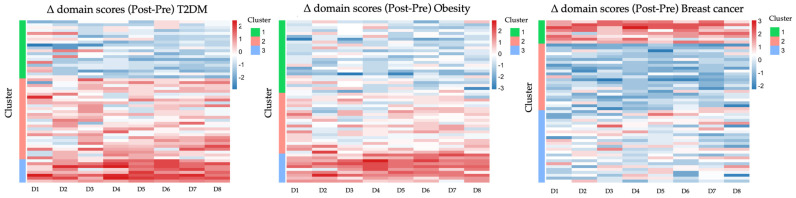
Heatmaps of domain-level pre–post score changes across three clinical scenarios. Each panel shows within-student change scores (post-test minus pre-test) for the eight diagnostic communication domains (D1–D8) in T2DM, obesity, and breast cancer (*n* = 56 students with complete data in all three scenarios). Rows represent individual students and columns represent domains. Cell colors indicate the magnitude and direction of score change, with values standardized within each scenario using z-scores to emphasize relative change patterns across domains. Students are grouped and ordered using k-means clustering (k = 3) based on their domain-level change profiles. Cluster labels are scenario-specific and reflect exploratory response-pattern groups rather than validated learner phenotypes or fixed low-to-high responder categories.

Substantial inter-individual heterogeneity in pre–post score change was observed. Heatmaps of domain-level gain scores (Figure 6) showed that positive score changes were not uniformly distributed across students or across domains within a given scenario. Rather than a single homogeneous pattern, students displayed variable combinations of larger, modest, and attenuated score changes depending on the clinical context. This heterogeneity was further explored using k-means clustering of three-scenario total-score gain profiles (Table 5). Using z-scored Δ total scores, a pragmatic k = 3 solution was used to describe exploratory response-pattern groups. Cluster A showed the largest mean score changes across scenarios, Cluster B showed intermediate changes, and Cluster C showed the smallest changes, including near-null or minimal changes in some contexts. Because clusters were derived from the same gain variables used to describe them, these between-cluster differences are presented descriptively and should not be interpreted as independent inferential evidence or as validated learner phenotypes.

## 4. Discussion

In this prospective single-arm pre–post educational study, completion of a structured generative AI-assisted simulation curriculum was associated with higher post-intervention diagnostic communication scores across three clinically distinct disclosure scenarios: T2DM, obesity, and breast cancer. Higher post-test scores were observed not only in the total rubric score, but also across all eight communication domains. However, the magnitude of pre–post score change was not uniform across scenarios or domains. The largest descriptive total-score change was observed in the breast cancer scenario, domain-level effects varied across clinical contexts, exploratory cross-scenario gain-score associations were limited and context-dependent, and individual pre–post change patterns were heterogeneous. Taken together, these findings extend the current literature by suggesting that AI-assisted communication simulation can be evaluated across multiple diagnostic disclosure contexts using standardized performance-based assessment, while also showing that score changes should not be assumed to generalize uniformly across diagnostic scenarios.

The most immediate implication of these findings is that AI-assisted simulation may be useful as a scalable adjunct for repeated diagnostic communication practice across more than one clinical context. Prior work in medical education has shown that communication competence can be strengthened through simulation-based training, but repeated exposure with individualized feedback is often constrained by the cost and logistics of standardized-patient programs [5,7,16]. Large language model-based systems offer an attractive complement because they permit high-frequency, low-marginal-cost rehearsal with immediate formative feedback [8,9,10,17,18]. The present findings are consistent with this rationale, as students demonstrated positive pre–post score changes across all three scenarios using a standardized rubric-based assessment. However, because the study lacked a control group, these changes cannot be attributed specifically to the AI-assisted component and may also reflect repeated exposure, rubric familiarization, concurrent clinical learning, or maturation. This distinction is important because much of the current literature on generative AI in health professions education remains descriptive, feasibility-focused, or based on self-reported outcomes rather than observed performance [8,9,10,19,20].

At the same time, the present results argue against treating diagnostic communication as a generic, scenario-independent competency. The pattern of pre–post score change depended on the clinical context. The breast cancer scenario showed the largest descriptive changes in total score and several individual domains, whereas the cardiometabolic scenarios showed smaller changes and different internal profiles. This scenario dependence is educationally plausible. Communicating a breast cancer diagnosis differs from counseling a patient with obesity or disclosing T2DM not only in emotional valence, but also in explanatory structure, stigma burden, decisional urgency, perceived threat, and the balance between empathic containment and structured information delivery [3,4,11,12]. These are not interchangeable communicative tasks. The present findings support the use of multi-scenario assessment when evaluating diagnostic communication training, particularly when the goal is to benchmark performance across clinically and emotionally distinct encounters.

Domain-level findings also have curricular implications. Larger score changes were concentrated in domains related to encounter organization and information exchange, including opening the discussion, gathering information, sharing information, and providing closure. In contrast, changes were smaller in the domain of understanding the patient’s perspective. This pattern suggests that some communication behaviors may be more readily shaped through repeated scripted practice and immediate feedback, whereas deeper patient-centered skills may require longitudinal coaching, reflective debriefing, supervised clinical exposure, and explicit attention to emotional and contextual complexity. AI-assisted simulation may therefore be most appropriately conceptualized as one component of a broader communication curriculum rather than as a replacement for faculty-led feedback or standardized-patient debriefing [6,7,20,21].

The exploratory cross-scenario analyses further reinforce the need for caution when interpreting gain scores as evidence of transferable competence. Gain-score associations were limited and context-dependent: changes in one scenario did not consistently correspond to changes in another. However, these correlations should not be interpreted as direct evidence of transfer or lack of transfer. Gain scores are sensitive to baseline performance, regression to the mean, ceiling effects, and measurement error. Therefore, the more defensible interpretation is that diagnostic communication performance should be assessed across multiple contexts rather than inferred from a single case. Future studies should use controlled longitudinal designs, residualized change models, and follow-up standardized-patient assessments to evaluate whether skills practiced in one diagnostic context are retained and applied to other contexts [12,13,17,22].

The heterogeneity analyses are particularly important in this regard. Heatmaps and responder clustering showed that improvement was not distributed along a simple linear continuum. Some students exhibited consistently strong gains across scenarios, others showed intermediate but broad improvement, and others demonstrated attenuated or context-specific gains. This pattern should not be dismissed as statistical noise. In educational terms, heterogeneity is itself information. It suggests that AI-supported communication training may function not only as an intervention, but also as a measurement-rich environment that can expose learner-specific profiles not easily visible in aggregate means. That possibility aligns with contemporary views of competency-based education, in which variability is not a nuisance to be averaged away, but a signal that should guide targeted remediation, progression decisions, and curricular design [6,7,19,21].

The use of generative AI in communication training also raises methodological and governance considerations. Although standardized prompts, fictional cases, faculty review of representative outputs, and privacy instructions were implemented, large language models remain probabilistic systems that may generate inconsistent, incomplete, biased, or clinically inaccurate responses. In this study, model-generated feedback was used only for formative practice and not as an authoritative clinical source or summative assessment tool. Future implementations should incorporate prospective monitoring of AI outputs, predefined procedures for identifying hallucinations or biased responses, transparent documentation of model version and access route, and clear data-protection safeguards. These requirements are particularly important when AI systems are used in sensitive communication scenarios involving stigma, cancer diagnoses, emotional distress, or potentially vulnerable learners.

This study has limitations. First, the single-arm pre–post design precludes causal attribution of the observed score changes to the AI-assisted simulation curriculum. Without a no-intervention, wait-list, active comparator, or non-AI simulation control group, alternative explanations cannot be excluded, including testing effects, repeated exposure to the assessment format, rubric familiarization, maturation, concurrent clinical learning, and regression to the mean. Second, assessments were scored live rather than from anonymized recordings presented in random order; therefore, complete blinding to the pre-test versus post-test phase could not be guaranteed, creating potential expectation bias. Third, participants were recruited from a single institution within one cultural and linguistic context, which may limit external generalizability. Fourth, although the use of three scenarios strengthens ecological and educational relevance, it also introduces complexity: diagnostic disclosure tasks are not emotionally or cognitively equivalent, and some observed variation may reflect scenario characteristics rather than differences in underlying communication competence. Fifth, despite standardized prompts and publicly available training materials, GPT-5 was accessed through the ChatGPT interface rather than a fully controlled API environment; therefore, model-level parameters and backend routing were not controlled by the investigators, limiting exact reproducibility. Sixth, individual student-level AI outputs were not exhaustively audited in real time, so undetected variability, hallucination, bias, or inconsistency in model responses cannot be excluded. Seventh, although the adapted rubric was reviewed by experts and inter-rater agreement was acceptable, further psychometric evaluation of the instrument across institutions and learner levels is needed. Finally, the study assessed short-term post-intervention performance in simulated encounters and did not evaluate long-term retention, transfer to real clinical encounters, patient outcomes, or effects on faculty workload and curricular implementation. Although several learner-level characteristics may plausibly influence pre–post score change, including baseline communication performance, prior LLM use, digital self-efficacy, empathy, communication-related self-confidence, academic performance, and prior clinical exposure, the present study was not powered for reliable multivariable predictor modeling. We therefore limited inferential interpretation to baseline–gain relationships and reported other learner characteristics descriptively. Future controlled studies with larger samples should examine predictors and moderators of response to AI-assisted simulation to determine which learners benefit most and which require additional faculty-led support.

Future research should move in three directions. The first is causal clarification: multi-scenario randomized trials are needed to determine the added value of AI-assisted simulation over conventional communication training, non-AI virtual patients, standardized-patient practice, and faculty-led feedback. The second is mechanism: studies should examine whether observed score changes are driven primarily by repetition, immediate feedback, scenario variability, learner motivation, baseline performance, or interactions among these factors. The third is implementation: future work should evaluate how AI-assisted simulation performs when embedded longitudinally within curricula, how it interacts with faculty-led debriefing, whether performance changes persist over time, and whether skills demonstrated in simulated encounters translate to later standardized-patient assessments or real clinical communication. The present study supports the rationale for such work by showing that AI-assisted simulation can be evaluated using multi-scenario, rubric-based performance assessment, while also underscoring that diagnostic communication remains context-dependent and educationally heterogeneous.

## 5. Conclusions

In this prospective single-arm pre–post educational study, generative AI-assisted simulation was associated with higher post-intervention diagnostic communication scores across T2DM, obesity, and breast cancer disclosure scenarios. Positive pre–post score changes were observed across total rubric scores and communication domains, but their magnitude and pattern varied by clinical context and by learner. Exploratory cross-scenario gain-score associations were limited and context-dependent, and clustering analyses suggested heterogeneous response-pattern groups rather than validated learner profiles. Because the study lacked a control group, these findings should be interpreted as within-student pre–post associations and not as causal evidence of AI-assisted simulation effectiveness. Multi-scenario evaluation may be useful when benchmarking diagnostic communication competence and when designing targeted remediation or longitudinal training pathways. Future controlled studies should clarify the added value of AI-assisted simulation over conventional instruction, non-AI virtual patients, standardized-patient practice, and faculty-led feedback, as well as its durability and transfer to real clinical communication.

## Figures and Tables

**Table 1:** Baseline sociodemographic, academic, and digital profile of enrolled medical students (*n*= 56).

Variable	Category/Units	N (%) or Mean ± SD
Age	Years	21.9 ± 1.5
Sex	Female	40 (71.40%)
	Male	15 (26.78%)
	Non-binary	1 (1.82%)
Current clinical cycle	Cycle III	56 (100.00%)
Extracurricular clinical employment/extra hospital shifts	Yes	3 (5.53%)
Cumulative GPA	0–10	8.45 ± 0.43
Prior formal coursework in diagnostic communication	Yes	0 (0.00%)
OSCEs completed	Median (IQR)	0 (0.00%)
Prior unsupervised real-patient care	Yes	39 (69.64%)
Prior communication of weight/obesity/metabolic-risk results	Yes	39 (69.64%)
Prior ChatGPT/LLM use (past 3 months)	Daily/almost daily	7 (12.50%)
	2–3 times/week	15 (26.78%)
	Weekly	22 (39.28%)
	Monthly	4 (7.14%)
	Less than monthly (1–2 times/3 months)	5 (8.92%)
	Never	1 (1.78%)
Digital self-efficacy	High (4–5)	20 (35.71%)
	Medium (3)	29 (51.78%)
	Low (1–2)	5 (8.92%)
Motivation to participate	1–5	4.09 ± 0.88
Self-confidence (1–5)	Explain lab results	3.9 ± 0.8
	Explain diagnostic criteria	4.0 ± 0.9
	Convey bad news empathically	3.9 ± 0.8
	Use teach-back	3.6 ± 0.9
	Reduce patient anxiety	3.7 ± 0.7
Jefferson Scale of Empathy (total)	20–140	124.1 ± 7.5

Digital self-efficacy and motivation were rated on 1–5 Likert scales (higher scores indicate greater self-efficacy/motivation). Self-confidence items were rated on 1–5 Likert scales (1 = very low, 5 = very high). Jefferson Scale of Empathy total was computed as the sum of 20 items (1–7), with reverse-coded items recoded prior to summation.

**Table 2:** Pre- and post-intervention diagnostic communication performance by Kalamazoo domains across three clinical scenarios.

Item	Domain	T2DM (*n* = 72) (Mean ± SD)		Obesity (*n* = 77) (Mean ± SD)		Breast Cancer (*n* = 67) (Mean ± SD)		Three-Scenario Completers (*n* = 56) (Mean ± SD)	
		Pretest	Post Test	Pretest	Post Test	Pretest	Post Test	Pretest	Post Test
1	Build a Relationship	8.51 ± 2.34	11.11 ± 3.01	7.36 ± 2.20	10.74 ± 2.42	5.87 ± 2.10	10.84 ± 2.26	7.28 ± 1.43	10.91 ± 1.70
2	Opening the Discussion	5.24 ± 1.52	9.40 ± 3.28	4.22 ± 1.07	7.51 ± 2.77	4.39 ± 1.31	9.00 ± 2.76	4.56 ± 0.89	8.61 ± 1.69
3	Gather Information	6.93 ± 2.23	10.11 ± 2.99	6.69 ± 2.03	9.86 ± 2.50	4.42 ± 1.62	10.79 ± 2.24	6.09 ± 1.21	10.24 ± 1.39
4	Understand the Patient’s Perspective	4.81 ± 1.75	6.97 ± 3.64	4.05 ± 1.32	6.82 ± 3.21	3.78 ± 1.28	6.28 ± 3.13	4.21 ± 0.91	6.47 ± 1.93
5	Share Information	5.47 ± 1.96	8.86 ± 3.51	5.17 ± 1.84	8.92 ± 2.91	4.03 ± 1.33	8.88 ± 2.89	4.83 ± 1.11	8.86 ± 1.84
6	Reach Agreement	6.33 ± 1.90	8.94 ± 3.59	5.91 ± 1.73	9.00 ± 3.07	4.34 ± 1.52	8.09 ± 2.37	5.42 ± 1.06	8.51 ± 1.73
7	Provide Closure	5.69 ± 2.10	9.12 ± 3.55	5.40 ± 1.80	8.61 ± 2.82	4.79 ± 1.59	9.73 ± 2.59	5.20 ± 1.16	9.07 ± 1.72
8	Scenario-specific Communication	5.99 ± 2.01	8.71 ± 3.72	6.03 ± 2.26	6.48 ± 2.05	5.84 ± 2.32	10.15 ± 2.26	5.84 ± 1.24	8.39 ± 1.58
Total	Overall score (24–120)	48.97 ± 9.92	73.24 ± 23.65	44.83 ± 9.03	67.94 ± 18.75	37.45 ± 9.91	73.76 ± 16.18	43.42 ± 6.13	71.08 ± 11.24

Domain scores are reported as domain totals (range 3–15) based on three items per domain. The total score represents the sum of all 24 items (range 24–120). Values are presented as mean ± standard deviation. Scenario sample sizes reflect participants who completed both the pre-test and post-test for each scenario (T2DM, obesity, and breast cancer).

**Table 3:** Within-student changes in diagnostic communication performance by Kalamazoo domains across three clinical scenarios.

Item	Domain	T2DM Δ (*n* = 72) (Mean ± SD)	Obesity Δ (*n* = 77) (Mean ± SD)	Breast Cancer Δ (*n* = 67) (Mean ± SD)	Three-Scenario Completers Δ (*n* = 56) (Mean ± SD)
1	Build a Relationship	2.60 ± 3.77 (1.71–3.48)	3.38 ± 3.00 (2.70–4.06)	4.97 ± 2.98 (4.24–5.70)	3.63 ± 2.04 (3.08–4.18)
2	Opening the Discussion	4.17 ± 3.33 (3.38–4.95)	3.29 ± 2.78 (2.66–3.92)	4.61 ± 2.92 (3.90–5.32)	4.05 ± 1.77 (3.58–4.53)
3	Gather Information	3.18 ± 3.49 (2.36–4.00)	3.17 ± 3.12 (2.46–3.88)	6.37 ± 2.76 (5.70–7.05)	4.15 ± 1.75 (3.69–4.62)
4	Understand the Patient’s Perspective	2.17 ± 4.27 (1.16–3.17)	2.77 ± 3.12 (2.06–3.48)	2.51 ± 3.20 (1.73–3.29)	2.26 ± 2.00 (1.73–2.80)
5	Share Information	3.39 ± 4.09 (2.43–4.35)	3.75 ± 3.43 (2.97–4.53)	4.85 ± 3.12 (4.09–5.61)	4.03 ± 1.97 (3.50–4.56)
6	Reach Agreement	2.61 ± 3.72 (1.74–3.48)	3.09 ± 3.35 (2.33–3.85)	3.75 ± 2.64 (3.10–4.39)	3.10 ± 1.76 (2.62–3.57)
7	Provide Closure	3.43 ± 3.99 (2.49–4.37)	3.21 ± 3.50 (2.41–4.00)	4.94 ± 2.83 (4.25–5.63)	3.88 ± 1.99 (3.34–4.41)
8	Scenario-specific Communication	2.72 ± 4.26 (1.72–3.72)	3.52 ± 3.51 (2.72–4.32)	4.31 ± 3.49 (3.46–5.16)	3.58 ± 2.12 (3.02–4.15)
Total	Overall score (24–120)	24.26 ± 25.05 (18.38–30.15)	26.17 ± 20.67 (21.48–30.86)	36.31 ± 17.70 (32.00–40.63)	28.68 ± 11.66 (25.56–31.81)

Notes: Domain scores are domain totals based on three items per domain (range 3–15; Δ range −12 to +12). The total score represents the sum of all 24 items (range 24–120; Δ range −96 to +96). Scenario-specific *n* values reflect participants with complete paired pre–post data for that scenario. The three-scenario completer column summarizes the mean within-student change averaged across the three scenarios (*n* = 56).

**Table 4:** Within-student gains across Kalamazoo domains among three-scenario completers (*n* = 56).

Item	Domain	Three-Scenario Completers Δ (*n* = 56) (Mean ± SD)	95% CI (Δ)	Cohen’s d	*p* (Holm-Adjusted)
1	Build a Relationship	3.63 ± 2.04	3.08–4.18	1.78	<0.0001
2	Opening the Discussion	4.05 ± 1.77	3.58–4.53	2.29	<0.0001
3	Gather Information	4.15 ± 1.75	3.69–4.62	2.38	<0.0001
4	Understand the Patient’s Perspective	2.26 ± 2.00	1.73–2.80	1.13	<0.0001
5	Share Information	4.03 ± 1.97	3.50–4.56	2.04	<0.0001
6	Reach Agreement	3.10 ± 1.76	2.62–3.57	1.76	<0.0001
7	Provide Closure	3.88 ± 1.99	3.34–4.41	1.94	<0.0001
8	Scenario-specific Communication	3.58 ± 2.12	3.02–4.15	1.69	<0.0001
Total	Overall score (24–120)	28.68 ± 11.66	25.56–31.81	2.46	<0.0001

Values represent within-student change (Δ = post-test − pre-test) averaged across the three scenarios for each student, then summarized across students (*n* = 56). Domain scores are domain totals based on three items per domain (range 3–15; Δ range −12 to +12). The overall score is the sum of all 24 items (range 24–120; Δ range −96 to +96). Cohen’s dz was computed as mean(Δ)/SD(Δ). *p*-values are from one-sample *t*-tests of Δ versus 0 with Holm correction across the nine outcomes (eight domains plus overall).

**Table 5:** Exploratory response-pattern groups based on three-scenario Δ total scores among three-scenario completers (*n* = 56).

Scenario	Cluster A (High Responder) Mean Δ ± SD (*n*)	Cluster B (Moderate Responder) Mean Δ ± SD (*n*)	Cluster C (Low Responder) Mean Δ ± SD (*n*)	*p*-Value *
T2DM	59.11 ± 12.44 (*n* = 9)	28.63 ± 5.24 (*n* = 27)	−1.55 ± 10.46 (*n* = 20)	<0.0001
Obesity	58.17 ± 11.48 (*n* = 12)	27.43 ± 7.87 (*n* = 23)	9.43 ± 10.16 (*n* = 21)	<0.0001
Breast cancer	64.00 ± 12.75 (*n* = 12)	33.58 ± 6.70 (*n* = 31)	16.08 ± 7.42 (*n* = 13)	<0.0001

Clusters were derived using k-means (k = 3) on z-scored three-scenario Δ total scores (Δ = post-test − pre-test) for T2DM, obesity, and breast cancer among participants completing all three scenarios (*n* = 56). Clusters were labeled A/B/C by descending mean Δ across scenarios. * The reported *p*-values are descriptive/exploratory because the same gain variables were used to derive and describe the clusters; therefore, between-group differences were not interpreted as independent inferential evidence.

## Data Availability

The datasets generated and analyzed during this study—including anonymized rubric scores, survey instruments, AI prompt templates, simulation scripts, and training materials—are publicly available in the Open Science Framework (OSF) repository at https://doi.org/10.17605/OSF.IO/X3UVW.

## Abbreviations

The following abbreviations are used in this manuscript:

AI	Artificial intelligence
CI	Confidence interval
CISESVI	Centro Internacional de Simulación y Entrenamiento en Soporte Vital Iztacala
IQR	Interquartile range
LLM	Large language model
OSF	Open Science Framework
SD	Standard deviation
T2DM	Type 2 diabetes mellitus
UNAM	Universidad Nacional Autónoma de México
URDM	Unidad de Remisión de Diabetes Mellitus
